# A3, a Scorpion Venom Derived Peptide Analogue with Potent Antimicrobial and Potential Antibiofilm Activity against Clinical Isolates of Multi-Drug Resistant Gram Positive Bacteria

**DOI:** 10.3390/molecules23071603

**Published:** 2018-07-02

**Authors:** Ammar Almaaytah, Ahmad Farajallah, Ahmad Abualhaijaa, Qosay Al-Balas

**Affiliations:** 1 Department of Pharmaceutical Technology, Faculty of Pharmacy, Jordan University of Science and Technology, Irbid 2110, Jordan; ahmad1990_frj@yahoo.com; 2 Department of Applied Biological Sciences, Faculty of Science and Arts, Jordan University of Science and Technology, Irbid 2110, Jordan; ahkhaled18@yahoo.com; 3 Department of Medicinal Chemistry, Faculty of Pharmacy, Jordan University of Science and Technology, Irbid 2110, Jordan; qabalas@just.edu.jo

**Keywords:** scorpion venoms, host defense peptides, antimicrobial activity, antibiofilm activity, synergism, bacterial resistance

## Abstract

Current research in the field of antimicrobials is focused on developing novel antimicrobial agents to counteract the huge dilemma that the human population is mainly facing in regards to the rise of bacterial resistance and biofilm infections. Host defense peptides (HDPs) are a promising group of molecules for antimicrobial development as they display several attractive features suitable for antimicrobial activity, including their broad spectrum of activity and potency against bacteria. AamAP1 is a novel HDP that belongs to the venom of the North African scorpion *Androctonus amoeruxi*. In vitro antimicrobial assays revealed that the peptide displays moderate activity against Gram-positive and Gram-negative bacteria. Additionally, the peptide proved to be highly hemolytic and displayed significantly high toxicity against mammalian cells. In our study, a novel synthetic peptide analogue named A3 was synthetically modified from AamAP1 in order to enhance its activity and toxicity profile. The design strategy depended on modifying the amino acid sequence of AamAP1 in order to alter its net positive charge, percentage helicity and modify other parameters that are involved theoretically in HDPs activity. Accordingly, A3 was evaluated for its in vitro antimicrobial and anti-biofilm activity individually and in combination with four different types of conventional antibiotics against clinical isolates of multi-drug resistant (MDR) Gram-positive bacteria. A3 was also evaluated for its cytotoxicity against mammalian cells. A3 managed to selectively inhibit the growth of a wide range of resistant strains of Gram-positive bacteria. Our results also showed that combining A3 with conventional antibiotics caused a synergistic antimicrobial behavior that resulted in decreasing the MIC value for A3 peptide as low as 0.125 µM. At the concentrations needed to inhibit bacterial growth, A3 displayed minimal mammalian cell toxicity. In conclusion, A3 exhibits enhanced activity and selectivity when compared with the parent natural scorpion venom peptide. The combination of A3 with conventional antibiotics could provide researchers in the antimicrobial drug development field with a potential alternative for conventional antibiotics against MDR bacteria.

## 1. Introduction

Bacterial infectious diseases represent a major health problem currently facing humanity and threatening the proper and effective treatment of several infectious diseases that are mainly caused by pathogens that have acquired the ability to resist all types of conventional antibiotics currently found in the clinic [1]. According to a recent World Health Organization (WHO) report regarding the emergence of bacterial resistance, the most notorious forms of bacteria causing the major types of resistant nosocomial infections were attributed to three distinct pathogens and they include *Escherichia coli*, *Klebsiella pneumoniae*, and *Staphylococcus aureus* [2].

The misuse and overuse of antibiotics by health practitioners over the recent decades has contributed significantly to the issue of microbial resistance [3]. Additionally, this sharp rise in antimicrobial resistance has also been accompanied by a short supply of novel classes of antibiotics being developed and reaching the clinic [4].

One of the most notorious infectious causing bacteria includes Gram-positive bacteria such as the multidrug-resistant *Staphylococcus aureus* and the multidrug-resistant *Enterococcus* spp. These pathogens are found in the hospital environment, and they exhibit a multidrug resistance mode of survival that complicates antimicrobial therapy. This continuous rise in antimicrobial resistance will lead to a major impact on the health of humans in addition to a huge barrier with health management costs and expenditure that will arise because of this dilemma [5].

Accordingly, as humans are set to face the post-antibiotic era if the current trend of microbial resistance continues at this pace, there is a strong global need for the development of new classes of antimicrobials that will offer humanity with different options to treat diseases that will be caused by these resistant pathogen [6].

Host defense peptides (HDPs) represent an integral component of the innate defense system of several organisms [7]. As part of the natural innate immune system, HDPs play a significant role in protecting these organisms from invasion or attack by bacteria, viruses, and fungi [8]. Most HDPs are composed of short peptide sequences with the average number of amino acids constituting these peptides not exceeding 50 amino acids on average. HDPs display a net positive charge and exhibit an amphipathic nature [9]. The net positive charge of HDPs along their amphipathic nature allow these peptide to interact with the negatively charged membranes of bacterial cells and damage the bacterial membrane causing cell lysis death eventually [10].

The main structural determinants that are responsible for defining HDPs’ membrane activity and are thought to influence the activity and toxicity of the HDPs are conformation or helicity, charge, hydrophobicity, hydrophobic moment, and amphipathicity. These factors cannot be assessed individually when trying to analyze the activity or toxicity of an antimicrobial peptide, as all factors are interactive and of significant importance [11,12].

Due to their attractive properties regarding the broad-spectrum antimicrobial activity and rapid killing kinetics, HDPs can be considered as potentially promising candidates for development of novel therapeutics against multi-drug resistant bacteria. HDPs may offer several advantages as candidates for antimicrobial development over traditional antibiotics as their use may include defeating infections individually or in synergy with other antimicrobial agents for the purpose of reducing the effective killing concentrations and consequently reducing cytotoxicity.

AamAP1 is a scorpion HDP that was identified through shotgun cloning from the venom of the North African scorpion *Androctonus amoeruxi*, the peptide carries a net positive charge of (+1) and was found to displays moderate activity against Gram-positive and Gram-negative bacteria [13].

In this study, we have analyzed the physiochemical properties of AamAP1 in order to modify these parameters for rational design of a novel synthetic peptide analogue for the purpose of enhancing the antimicrobial activity and reducing cell cytotoxicity. The design strategy employed in this study depended on modifying the net positive charge of the natural peptide while keeping other physiochemical parameters within normal ranges. The aim of the design strategy was focused on producing a modified peptide with enhanced and potent activity against clinical isolates of multidrug-resistant Gram-positive bacteria of the *Staphylococcus aureus* and *Enterococcus* spp. Additionally, and in order to reduce the minimum effective antibacterial concentrations of the modified peptide and consequently reduce its toxicity on mammalian cells, we combined the modified peptide named A3 with four conventional antibiotics to enhance the activity of each individual antimicrobial agent through a synergistic mode of action. Additionally, the anti-biofilm activity of A3 was evaluated against biofilm-forming Gram-positive bacteria. Finally, the in vitro cytotoxicity of A3 against normal mammalian cell lines in addition to human erythrocytes was evaluated in order to assess the outcome of the design strategy and the synergistic mode of action in the reduction of HDPs’ toxicity.

## 2. Materials

### 2.1. Bacterial Strains

In the present study, the Gram-positive bacterial strains used for the determination and testing of the antimicrobial activity of A3, the antibiotics, and A3-antibiotic combinations were acquired from the American type tissue culture collection (ATCC) and these include: *Staphylococcus aureus* (ATCC 29213), *Enterococcus faecalis* (ATCC 29212), *Enterococcus faecalis* (ATCC 19433), and *Staphylococcus epidermidis* (ATCC 12228) which were used as control strains. Additionally, the multi-drug resistant bacteria including methicillin-resistant *Staphylococcus aureus* (MRSA) (ATCC 33591), (ATCC 43300), and (ATCC BAA-41), and the multi-drug resistant *Enterococcus faecalis* (ATCC BAA-2356) and *Enterococcus faecium* (ATCC BAA-2316) were also employed in our tests. The bacteria was cultured on Mueller Hinton Agar obtained from Scharlab, S.L. (Barcelona, Spain).

### 2.2. Antimicrobial Substances

Different antimicrobial substances were employed in the study, including AamAP1, A3, and four kinds of conventional antibiotics that include levofloxacin, chloramphenicol, rifampicin, and erythromycin.

AamAP1 and its modified analogue (A3) were synthesized by employing the standard solid phase chemistry of peptide synthesis. Dimethylformamide (DMF) was used a the solvent for the peptide synthesis while 2-(1*H*-Benzotriazole-1-yl)-1,1,3,3-tetramethyluronium hexafluorophosphate (HBTU) was used as an activator. Fmoc was deprotected by 20% piperidine, and Wang resin was cleaved using trifluoroacetic acid (TFA). The purification of the peptides was performed by reverse-phase high performance liquid chromatography (RP-HPLC) using an acetonitirile/H_2_O-TFA gradient. Electron spray ionization mass spectrometry was employed for the identification of the peptide (GL Biochem Ltd., Shanghai, China).

All antibiotics were obtained from Sigma-Aldrich (St. Louis, MO, USA). Stock solutions were prepared according to the manufacturer’s recommendation and stored at optimum temperature for each antibiotic.

The media used to dissolve the peptide and the antibiotics and also to prepare bacterial suspensions was Mueller-Hinton broth obtained from Oxoid Ltd., Basingstoke, UK).

### 2.3. Cell Lines

The cell lines employed for the toxicity assays were the Human Embryonic Kidney 293 cells line (HEK 293) and Vero cell line. The culture media employed for cell propagation include RPMI media with 1% streptomycin and ampicillin and 10% fetal Bovine serum (St. Louis, MO, USA).

## 3. Methods

### 3.1. Bioinformatics Analysis and Design of the Modified Peptide

The network protein sequence analysis SOPM secondary structure prediction software was used for estimation of the α-helical content of AamAP1 and the modified peptide A3 in order to control the helicity parameter of the modified peptide within optimum range [14]. The mean hydrophobicity (hydrophobicity <H>, hydrophobic moment <µH>), was calculated using the HydroMCalc software for AamAP1 and A3 while Innovagen’s peptide calculator was used for calculating all other physicochemical parameters of both peptides.

### 3.2. Molecular Modeling and In Silico Analysis of AamAP1 and Its Modified Peptide Analogue A3

The chemical properties AamAP1’s structural analogue A3 were analyzed through computer simulation. Identification and structure prediction for the best template for homology modeling of A3 was performed using the HHpred (HHsearch 2.0) software by HMM–HMM comparison [15]. The RAMPAGE software was employed to validate the 3D model produced by homology modelling [16]. The Accelrys^®^ Discovery studio software was used for A3’s model visualization and the I-TASSER software was employed to confirm the reliability of the produced model [17].

### 3.3. Minimum Inhibitory Concentrations (MIC) and Minimum Bactericidal Concentrations (MBC)

The minimum inhibitory concentrations (MICs) and the minimum bactericidal concentrations (MBCs) of A3 against the bacterial strains were determined by adapting the microbroth dilution method outlined by the Clinical and Laboratory Standards Institute (CLSI) guidelines [18,19]. MHB was employed for bacterial propagation overnight and the bacteria finally diluted to 10^6^ CFU/mL. Different concentrations of A3 were prepared by using the serial dilution method in culture medium. Once the peptide–bacteria combinations were prepared in the 96-well plates, the plates were incubated at 37 °C for 24 h in a humidified incubator. Optical density (OD) at λ = 600 nm was employed for the determination of bacterial growth using an ELISA reader (BioTek Epoch, Winooski, VT, USA). For the minimum bactericidal concentration (MBC), 10 μL aliquots were taken from wells of each peptide concentration, then transferred into a pre-sterilized labeled agar plates and incubated over night at 37 °C for overnight according to CLSI guidelines. The MBC was determined as the lowest concentration that resulted in <0.1% survival of the subculture.

### 3.4. MIC and MBC Determination of Individual Antibiotics and Checkerboard Assay

The (MICs) and (MBCs) of antibiotics were tested and determined against bacterial strains as described in the previous section by preparing eight different concentrations of each antibiotic. The (MICs) and (MBCs) of combinations of A3 and the different antibiotics were tested and determined against bacterial strains as described in Section 3.3 in addition to the broth microdilution checkerboard technique [20]. A mixture of the peptide and one of the antibiotics in different concentrations were added to each microtiter well, where 25 µL of each peptide concentration and 25 µL of each antibiotic concentration were added to six wells of a sterile flat-bottomed 96-well plate containing 50 µL of diluted bacterial suspension. All MIC and MBC determinations were made in triplicate. 

### 3.5. Synergistic Studies Evaluation and the Fractional Inhibitory Concentration (FIC)

The checkerboard techniques was used for determining the FIC for A3 and its antibiotic combinations [20]. The FIC is defined as the inhibitory concentration of the antimicrobial combination divided by that of the single antimicrobial component. The FIC index for the combination of different two antimicrobial agents is calculated according to the following equation:

FIC index = (MIC of drug X in combination)/(MIC of drug X alone) + (MIC of drug Y in combination)/(MIC of drug Y alone). FIC indices were interpreted as follows: ≤0.5: synergistic activity, 0.5–1: additive activity, 1–4: indifference, >4: antagonism.

A synergistic activity means that each individual component in the combination is supplementing the other in increasing its potency. Additive activity represents an increase in the potency of only one component in the combination, while indifference means there is no change in the activity between individual and combination treatments. Finally, antagonistic activity means that one or even both components in the combination are working against each other.

### 3.6. Antibiofilm Activity

The anti-biofilm activity for A3 and was performed according to Luca et al. [21]. The Calgary biofilm device (Innovotech, Edmonton, AB, Canada) was employed for forming biofilms of *S. aureus* (29213) and *S. aureus* (BAA-41) bacterial strains. A 10^7^ CFU/mL concentration of each bacterial strain was achieved by propagating the bacterial cells overnight in MHB followed by dilution. For biofilm formation, the bacterial cells were grown on the Calgary peg lids for biofilm buildup and growth followed by incubation for 20 h under a rotation of 125 rpm at 35 °C. The negative control was established by adding MHB instead of the bacteria to the peg designed wells. The Calgary device allows the determination of the minimum biofilm eradication concentration (MBEC) which corresponds to the lowest antimicrobial concentration capable of inhibiting bacterial regrowth after antimicrobial biofilm exposure. Each peg-lid was then transferred into a “challenge 96-well microtiter plate” containing two hundred microliters of different peptide concentrations and the peg lids containing the biofilms were incubated for two hours at 37 °C. After biofilm treatment with the challenge plate, the biofilms were transferred into a recovery plate and incubated for eight hours. The MBEC of the each antibiotic was determined as described previously by preparing eight antibiotic solutions with different concentrations.

For viable cell determination, 5 µL from each well of peptide concentrations was transferred to MHB and then transferred into a pre-sterilized labeled agar plates and incubated over night at 37 °C. The following day, the number of bacterial colonies was counted. The minimum bactericidal concentration for biofilm (MBCb) was defined as the lowest peptide concentration that showed no growth (99.9% killing).

### 3.7. Mammalian Cytotoxicity Assays

The MTT assay was employed for the cytotoxicity assays, the adherent kidney cell lines (VERO and HEK) were seeded in 5 × 10^3^ cells per well in 96 well cell culture plates followed by incubation at 37 °C under 5% CO_2_ for 24 h. Different concentrations of A3 were added to each well and incubated as mentioned previously. After the incubation period and peptide treatment, each well was exposed to 30 μL MTT solution followed by 4–6 h of incubation. The plates were then removed and the MTT solution was replaced by DMSO to dissolve the formazan crystals. The plates were then placed on an ELx808™ absorbance microplate reader (BioTek, Winooski, VT, USA) and the absorbance measured at λ = 600 nm.

### 3.8. A3’s Hemolytic Activity

A3’s hemolytic activity was performed as described previously [13]. Briefly, human erythrocytes were exposed to different concentration of A3 to assess the percentage of hemolysis. Positive controls consisted of RBCs exposed to Triton X100 while the negative controls consisted of RBC suspension solely.

## 4. Results

### 4.1. Bioinformatics Analysis of AamAP1 and the Design of A3

Modification of the amino acid sequence of the scorpion venom peptide AamAP1 in order to increase its overall net positive charge along its helicity was the main objective of A3’s design strategy. Accordingly, the NPS SOPM software was used to determine the overall percentage helicity of the parent peptide that was subjected to amino acid substitution either with lysine or arginine in order to increase the net positive charge and ensure a boost in the helicity of the modified peptide. Other parameters related to hydrophobicity and hydrophobic moment were not significantly altered as a result of this substitution. The rationale behind increasing the positive charge and overall helicity of the parent peptide is related to HDPs mode of action. HDPs initially interact with the negatively charged bacterial membranes that is followed by the peptide’s ability to insert itself into bacterial membranes which is highly dependent of the net positive charge and helicity. Consequently, modifying these parameters would alter the mode of action of HDPs and their bacterial selectivity. As shown in Table 1, A3 was designed to display an extra two charges by substituting the proline with arginine and histidine with lysine on positions 7 and 8 respectively.

The results shown in (Table 1) display that parent peptide AamAP1 exhibits 55.56% helicity, while the structural modification performed on the parent peptide increased the percentage helicity of A3 to 100%. The hydrophobicity range generated for AmaAP1 was reported to be (0.904) while for A3, it was reported to be (0.746). The hydrophobic moment was also simulated for each peptide with a reported value of (0.435) for the AamAP1 and a value of 0.517 for A3.

**Figure 1:**
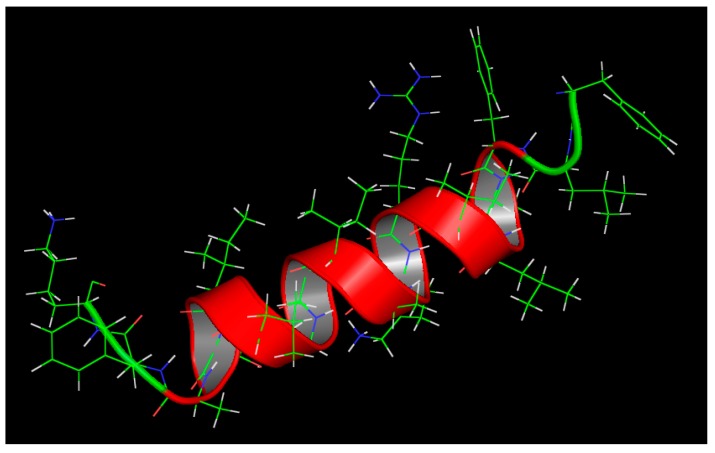
Accelrys^®^ Discovery Studio A3’s three dimensional model visualization. The visualization clearly indicates a continuous uninterrupted alpha helical structure.

The most optimal template for homology modeling of A3 and its structure identification were generated through the HHpred (HHsearch 2.0, Tubingen, Germany). The homology prediction reported that A3 displays good alignment with Pardaxin (an antimicrobial and anticancer peptide identified previously from Pacific Peacock sole). The homology score reported for A3 was 21.8, which is considered to be of high quality. Three-dimensional structural modeling of A3 revealed that the A3 is exhibiting a continuous uninterrupted alpha helix confirmation in accordance with the theoretical calculations performed previously (Figure 1).

### 4.2. Peptide Synthesis and Purification

A3 was synthesized to 95% purity as indicated by RP-HPLC purity (Figure S1, Supplementary Material). The mass spectrometry data confirmed the identity of A3 as it showed major peaks in the +2 and +3 charge state of 991.64 Da and 661.37 Da respectively (Figure S2, Supplementary Material). 

### 4.3. Bacterial Susceptibility Assay

A3 proved to be a highly active antimicrobial agent against all the studied bacterial strains. It was able to inhibit the growth of control strains of Gram-positive bacteria within a range of 2.5–12.5 µM. Additionally, it inhibited the growth of clinically isolated resistant Gram-positive strains within a range of 5–15 µM. *S. epidermidis* (12228) was the most sensitive strain with a MIC value of 2.5 µM.

The bactericidal activity of A3 peptide was assessed by measuring the minimal bactericidal concentration (MBC) for each tested bacterial strain. The MBC values reported for A3 peptide against all studied bacterial strains were equal to the MIC values which indicate that the peptide is exhibiting a bactericidal antimicrobial nature. The MIC and MBC values of A3 against the bacterial strains are summarized in Table 2.

The antimicrobial activities of the antibiotics employed in the current study were determined against wild type and multi-drug resistant bacterial strains. The MIC values of all antibiotics against the bacterial strains are summarized in Table 3. Four different types of antibiotics were included in the current study including levofloxacin, chloramphenicol, rifampicin, and erythromycin. Five strains of bacteria were selected for the determination of the MICs in regards to the previously mentioned antibiotics. One from the control group, represented by *S. aureus* (29213) and four strains representing the MDR group and these include *S. aureus* (33591)*, S. aureus* (BAA-41), *E. faecalis* (BAA-2356), and *E. faecium* (BAA-2316).

### 4.4. Synergistic Activity of A3 and the Antibiotics in Combination

The MIC values for A3 in combination with the antibiotics in most combination groups decreased dramatically. Eight combinations out of the overall 16 antimicrobial combinations displayed synergistic effects against planktonic cells of the tested bacterial strains. For *S. aureus* (ATCC: 29213), A3-levofloxacin, A3-chloramphenicol, A3-rifampicin, and A3-erythromycin displayed potent synergistic activities as the MIC of A3 in the first two combinations was reduced by 60% and in the last two combinations, the reduction in MIC was 97.5%. Levofloxacin’s MIC in the combination was reduced by 93.6%, chloramphenicol’s MIC by 93.8%, rifampicin’s MIC by 60%, and finally erythromycin by 87.5% when compared to the MICs of these antibiotics individually. For the MRSA strain of *S. aureus* (ATCC 33591), A3-levofloxacin and A3-rifampicin combinations displayed significant synergistic effects. The MIC of A3 in these combinations was reduced by 70% and 60% respectively when compared to its individual MIC. The MIC values of levofloxacin and rifampicin in the combination groups were reduced by 92.5% and 96.9% respectively compared to the MICs of these antibiotics individually (Table 4).

For the clinically isolated MRSA strain of *S. aureus* (ATCC BAA-41), only the A3-chloramphenicol combination displayed a synergistic effect. The MICs of A3 and chloramphenicol in the combination were reduced by 60% and 90% respectively compared to their individual MICs. For the clinically isolated multi-drug resistant *E. faecium* (BAA-2316), the A3-levofloxacin combination displayed a synergistic effect. The MICs of A3 and Levofloxacin in the combination were reduced to 86.7% and 90% respectively compared to their individual MICs.

### 4.5. The FIC Index

The FIC index results show that several A3-antibiotic combinations display potent synergistic effects with (FIC ≤ 0.5) against target bacteria. The combination of A3-erythromycin against *S. aureus* (29213), A3-levofloxacin against *E. faecium* (BAA-2316) and A3-levofloxacin against *S. aureus* (33591) exhibited the strongest synergistic combinations with FIC indices reaching 0.15, 0.23, and 0.38 respectively. The combinations of A3-levofloxacin, A3-chloramphenicol, and A3-rifampicin against *S. aureus* (29213) exhibited synergistic activity with the FIC indices reaching 0.46, 0.46, and 0.43 respectively. The combination of A3-Rifampicin against *S. aureus* (33591) displayed a synergistic activity with the FIC index reaching 0.43. The combination of A3-chloramphenicol against *S. aureus* (BAA-41) exhibit synergistic activity with the FIC indices reaching 0.5. The remaining combinations displayed additive effects with FICs in the range of (0.5 < FIC < 1). All The FIC indices of the A3-antibiotic combination groups are listed in Table 5.

### 4.6. Antibiofilm Activity

The antibiofilm activities of A3 and AamAP1 were assessed by two independent methods using the Calgary biofilm device. The first method depends on visual observation of bacterial growth and the determination of the minimal biofilm eradication concentration (MBEC), while the second method depends on viable bacterial cell counts after the treatment using the colony count method. The minimum A3 concentration that was able to inhibit the re-growth of bacteria from peptide treated of *S. aureus* (29213) and *S. aureus* (BAA-41) biofilms (MBEC) was found to be 25 and 30 µM respectively. There was no biofilm activity seen for the AamAP1, as shown in Table 6.

The minimum concentration of A3 needed to reduce the number of viable bacterial cells of *S. aureus* (29213) and *S. aureus* (BAA-41) biofilms to almost zero (99.9% killing) (MBCb) was calculated and the results of the minimum bactericidal concentration (MBCb) assay are summarized in Table 7. A3 managed to reduce the number of viable bacterial cells to almost zero and achieve the minimum bactericidal concentration (MBCb) at 60 µM which confirms the potent activity of the peptide and further increases the spectrum of activity of A3 to include bacterial biofilms in addition to the clinically isolated resistant strains of Gram-positive bacteria.

### 4.7. MTT Cell Proliferation Assay

The cell proliferation assay was performed for the purpose of measuring the antiproliferative activity of A3 against two types of mammalian cell lines (Vero and HEK 293) in order to measure the peptide selectivity and cytotoxicity. A3 managed to inhibit the proliferation of Vero and HEK cell lines at IC_50_ values of 26.1 and 33.2 µM respectively (Table 8).

### 4.8. Hemolysis Assay

The hemolytic activity of A3 against human erythrocytes (RBC) was determined as measure of the peptide’s toxicity toward normal mammalian cells. At the MIC and MBC concentrations for A3 in combination with the antibiotics that were reported to be in the range of (0.025–3 µM) in addition to the individual MIC concentrations of A3 against most bacterial strains evaluated previously. A3 caused almost zero (negligible) hemolysis after 60 min of incubation with human erythrocytes while significant hemolysis to human erythrocytes was reported with concentrations above 20 µM, which are significantly higher than the concentrations needed to inhibit bacterial growth individually, or with the antibiotic combinations (Table 9).

## 5. Discussion

HDPs are considered one of the few promising groups that are currently available for development into effective antimicrobials to combat infections caused by resistant bacteria [22]. However, as these agents display significant toxicity against mammalian cells and suffer from the lack of bacterial selectivity, the clinical development of these agents had been slow with no successful accomplishments or outcomes. Several HDPs display potent antimicrobial activity but suffer from severe toxicity and hemolytic activity against normal cells. To overcome this dilemma, many studies focused on advising new methods to limit the cytotoxicity of HDPs either through structural modification or novel formulation technologies [23,24,25].

Scorpion venoms represent a cocktail of biologically active molecules that are classified into two groups and these include the disulfide-bridged peptides that target membrane bound ion channels and the recently discovered non-disulfide bridged peptides which display a diversity of biological activities including the antimicrobial peptides [25,26]. As with other HDPs, scorpion antimicrobial peptides reported in literature share the same drawbacks in regards to the toxicity and lack of cell selectivity that is shared among this group of molecules [27]. AamAP1, a novel HDP that was identified from the venom derived cDNA library of the North African scorpion *Androctonus amoeruxi*, displays weak antimicrobial activity with MIC values in the range of 20–150 μM. The peptide is also strongly hemolytic and exhibits significant toxicity and a lack of selectivity against mammalian cells [13].

In the present study, AamAP1 was used as platform for computer aided rational design in order to develop a modified peptide with improved antimicrobial activity combined with improved cell selectivity and an overall decrease in toxicity against mammalian cells. The design strategy was mainly based on substitution of different amino acid in order to increase the positive net charge and the overall percentage helicity while taking into account the optimization of all the other physicochemical parameters responsible for HDPs activity. The main parameter that was initially employed for modification of the parent peptide focused on increasing the net positive charge and the generation of a continuous helical amino acid sequence. The resultant modified peptide named A3 was later screened for all the structural parameters involved in HDPs activity such as hydrohobicity, hydrophobic moment, charge, and percentage helicity

The overall net charge of HDPs is considered an essential parameter regarding HDPs activity. Charge is responsible for the initial electrostatic attraction of cationic HDPs with the negatively charged bacterial membranes. The increase in A3’s net positive charge while not drastically altering other physicochemical parameters is expected to enhance the peptide’s antimicrobial activity and minimize its cytotoxicity by enhancing its selectivity against bacterial membranes. An overall net charge range of (+2 < Q < +9) has been determined in literature as an optimal range for HDPs activity [28]. A3 displayed a positive cationic charge of (+3). When compared to the parent peptide AamAP1 that originally displayed a net charge of (+1).

The α-helical content and the percentage helicity of A3 was evaluated in order to confirm that the substitution design generated a continuous uninterrupted α-helical peptide. The results displayed that the percentage helicity of A3 was 100% with a significant increase over the parent peptide that exhibited 50.56% percentage helicity. As helicity is considered crucial for HDP activity, this increase in helicity in the modified peptide is expected to generate modified peptide with significant selectivity against target cells.

In order to confirm the initial helicity analytical results, the HHpred (HHsearch 2.0) software was used for homology modeling and for identifying the best-fit peptide for structure visualization and identification of the modified peptide A3. The homology results displays significant alignment with Pardaxin, a previously identified HDPs with potent antimicrobial and anticancer activities and an uninterrupted helical structure that is in accordance with initial helicity findings that were generated from the SOPM secondary structure prediction software. Previously, we have employed the same design methodology to design an enhanced peptide using the same natural scorpion peptides as template for rational design. The resultant peptide analog named AamAP1-lysine displayed enhanced antimicrobial activity with moderate mammalian cell toxicity. Additionally, mechanistic studies confirmed that AamAP1-lysine was able to lyse bacterial membranes and inhibit DNA gel retardation. However, these studies did not study the antimicrobial effect of such design strategy on clinical isolates of multidrug resistant bacteria or biofilms. The synergistic mode of action of AamAP1-lysine was also not investigated. Accordingly, this study aims to confirm the design strategy and explore the antibiofilm and synergistic activity of combining such designed peptides [29].

The antimicrobial studies displayed that A3 was active against the wild type and the multi-drug resistant clinical isolates of Gram-positive bacteria that were employed in this study. A3 was able to inhibit the growth of control strains of Gram-positive bacteria within a range of 2.5–12.5 µM. Additionally, it inhibited the growth of the multi-drug resistant and clinical isolates of Gram-positive strains within a range of 5–15 µM. The synergistic effects of combing A3 with conventional antibiotics was also evaluated in order to further reduce the effective antimicrobial concentration needed for bacterial elimination. The results from the synergistic studies display that the MIC values for a significant number of antibiotics in combination with A3 decreased dramatically. Out of the overall sixteen antimicrobial combinations, 50% were shown to be synergistic and 50% are additive against the planktonic cells of the tested bacterial strains while non-shown any indifference or antagonistic effect according to FIC index. For the control strain of *S. aureus* (ATCC: 29213), A3-levofloxacin, A3-chloramphenicol, A3-rifampicin, and A3-erythromycin combinations displayed synergistic effects. The most notable reduction was related to levofloxacin’s MIC reduction when combined with A3 as its MIC value was reduced by 93.6%. Additionally, chloramphenicol’s MIC value was reduced by 93.8%, while erythromycin’s MIC was reduced by 87.5% compared to the MICs of these antibiotics individually. 

For the MRSA strain of *S. aureus* (ATCC 33591), A3-levofloxacin and A3-rifampicin combinations displayed synergistic effects. The MIC values of levofloxacin and rifampicin in the combination groups were reduced by 92.5% and 96.9% respectively compared to the MICs of these antibiotics individually. For the clinical isolated MRSA strain of *S. aureus* (ATCC BAA-41), only the A3-chloramphenicol combination displayed a synergistic effect. The MIC of chloramphenicol in the combination was reduced to 90% compared to its individual alone. For the clinical isolated multi-drug resistant *E. faecium* (BAA-2316), A3-levofloxacin combination displayed a synergistic effect and the MIC of levofloxacin in the combination was reduced to 90% compared to its MIC alone.

Conventional antibiotics’ mode of eliminating bacteria depends on a well-defined interference with bacterial molecular targets that are related to cell growth and replication, these targets include inhibiting the bacterial cell wall synthesis or other intracellular mechanisms such as interfering with protein synthesis or DNA replication [30]. The synergistic and additive effects between A3 and the conventional antibiotics clearly display that both classes of molecules augment the activity of each individual antimicrobial agent. However, the exact mechanism of synergism between both agents remain to be elucidated. Several studies showed that many HDPs exhibit the ability to lyse the biological membrane and generate pores by different mechanisms such as toroidal pore and barrel stave models which consequently allow the antibiotics to bypass the bacterial cell wall in large numbers and destroy the bacteria [31].

It is well-documented that bacterial membranes display a net negative charge on their membranes while normal mammalian cells are usually zwitterionic. This relative increase in bacterial membrane negative charge is due to the presence of significant amounts acidic phospholipids peptidoglycans (PG), phospholipids (PS), and cardiolipins (CL) [12]. One of the proposed mechanisms for the synergistic effect is that the HDPs cause destruction of the peptidoglycan layer and cause permeabilization of the membrane and consequently allowing the rapid entry of antibiotics [32]. From our data, we propose that this mechanism is the most probable mechanism for A3 and the tested antibiotics combinations. This mechanism explains our results where all combinations displayed either a synergistic or additive effects against all tested bacterial strains. The targets of all antibiotics employed in this study (levofloxacin, chloramphenicol, rifampicin, and erythromycin) are located inside the bacterial cell, which means this mechanism facilitated the intracellular entry of antibiotics in reaching their targets and accomplishing their molecular function.

The structural modification not only enhanced the antimicrobial activity of the parent peptide but also supplemented it with potent antibiofilm activity. The minimum A3 concentration that was able to inhibit the re-growth of the clinically isolated multi-drug resistant Gram-positive bacteria of *S. aureus* (ATCC: 29213 and BAA-41) from peptide treated biofilm was found to be 25 µM and 30 µM, respectively. For the four conventional antibiotics used in our study levofloxacin, chloramphenicol, rifampicin, and erythromycin, the reported data for biofilm eradication was above 500 µM for each antibiotic (data not shown). This data suggests that A3 is highly potent agent in biofilm inhibition unlike conventional antibiotics. The increase in the cationicity and overall helicity of A3 not only enhanced its antimicrobial activity but also influenced its selectivity index. The design strategy employed in this study managed to increase the antimicrobial activity of A3 while lowering its hemolytic and toxic activity at the effective antimicrobial concentrations.

The reduced hemolytic activity of A3 correlated with the results from the MTT cytotoxicity assay against human HEK 293 and Vero cell lines. When compared with its parent peptide, A3 displayed minimal hemolysis against human RBCs. This behavior is probably related to the implemented modifications related to the net charge and helicity of the modified peptide which allowed the peptide to bind preferentially to bacterial membranes. The anti-proliferative concentrations of A3 was reported to be five- to six-fold higher than the geometric average MIC values which confirm that the design strategy was successful in reducing the overall toxicity of the peptide

In conclusion, we report the design of a modified synthetic analogue of the natural scorpion venom peptide AamAP1. The modified analogue named A3 displayed potent antimicrobial and antibiofilm activity against standard and MDR Gram-positive bacteria. A3 was also combined with conventional antibiotics in order to evaluate its synergistic activity and the results proved that high synergism occurs between A3 and several antibiotics against different strains of bacteria. Additionally, the design strategy, which focused on increasing the antimicrobial activity, reducing mammalian cell cytotoxicity and enhancing selectivity of A3, proved to be highly effective as A3 proved to exert minimal cytotoxic and hemolytic effects at the concentrations needed to inhibit bacterial growth. The combination of A3 with conventional antibiotics could prove to be of high value and a potential alternative for conventional antibiotics against MDR bacteria.

## Figures and Tables

**Table 1:** Amino acid sequence and physico-chemical parameters of both AamAP1 and its synthetic analogue A3.

Peptide Name	Amin Acid Sequence	Hydrophobicity (H)	Hydrophobic Moment (µH)	% Helicity	Net Charge z
AamAP1	FLFSLIPHAIGGLISAFK	0.904	0.435	55.56%	+1
A3	FLFSLIRKAIGGLISAFK	0.746	0.517	100%	+3

**Table 2:** Summary of the MIC and MBC values of A3 against all tested bacterial strains. Results represent triplicates.

**Control Gram-Positive Strains**	**ATCC**	**MIC Value (µM)**	**MBC Value (µM)**
*Staphylococcus aureus*	29213	5	5
*Enterococcus faecalis*	29212	10	10
*Enterococcus faecalis*	19433	12.5	12.5
*Staphylococcus epidermis*	12228	2.5	2.5
**MDR Gram-Positive Strains**	**ATCC**	**MIC Value (µM)**	**MBC Value (µM)**
*Staphylococcus aureus*	43300	5	5
*Staphylococcus aureus*	BAA-41	5	5
*Staphylococcus aureus*	33591	5	5
*Enterococcus faecalis*	BAA-2356	5	5
*Enterococcus faecium*	BAA-2316	15	15

**Table 3:** Minimum inhibitory concentrations (MICs) of antibiotics against the tested bacterial strains. MIC values are displayed in (µM) concentration. Results represent triplicates.

Antibiotic	*S. aureus* (29213)	*S. aureus* (33591)	*S. aureus* (BAA-41)	*E. faecalis* (BAA-2356)	*E. faecium* (BAA-2316)
Levofloxacin	0.5	10	10	27.5	12.5
Chloramphenicol	20	130	25	30	20
Rifampicin	0.025	0.04	0.005	0.03	7.5
Erythromycin	0.5	8	350	35	40

**Table 4:** Synergistic activity of A3 in combination with conventional different strains of standard and MDR bacteria. Results represent triplicates.

MIC in Combination against Gram-Positive Bacteria MIC in Combination/(Individual MIC)			
Bacterial Strain	Antibiotics (µM)		A3 (µM)
*S. aureus* (29213)	Levofloxacin	0.03215/(0.5)	2/(5)
	Chloramphenicol	1.25/(20)	2/(5)
	Rifampicin	0.01/(0.025)	0.125/(5)
	Erythromycin	0.0625/(0.5)	0.125/(5)
*S. aureus* (33591)	Levofloxacin	0.75/(10)	1.5/(5)
	Chloramphenicol	30/(130)	2.5/(5)
	Rifampicin	0.00125/(0.04)	2/(5)
	Erythromycin	3/(8)	2/(5)
*S. aureus* (BAA-41)	Levofloxacin	0.7/(10)	3/(5)
	Chloramphenicol	2.5/(25)	2/(5)
	Rifampicin	0.0002/(0.005)	3/(5)
	Erythromycin	50/(350)	2/(5)
*E. faecium* (BAA-2316)	Levofloxacin	1.25/(12.5)	2/(15)
	Chloramphenicol	10/(20)	2/(15)
	Rifampicin	6.25/(7.5)	2.5/(15)
	Erythromycin	30/(40)	1/(15)

**Table 5:** List of all the A3-antibiotic combinations and their FIC indices. Results represent triplicates.

FIC Index				
Antimicrobial Combinations	*S. aureus* (29213)	*S. aureus* (33591)	*S. aureus* (BAA-41)	*E. faecium* (BAA-2316)
A3-kevofloxacin	0.46	0.38	0.68	0.23
A3-chloramphenicol	0.46	0.73	0.5	0.63
A3-rifampicin	0.43	0.43	0.64	1.0
A3-erythromycin	0.15	0.78	0.54	0.82

**Table 6:** MBEC values of A3 and AamAP1 against biofilm-forming bacterial strain of *S. aureus.* MBEC values are displayed in (µM) concentration. Results represent triplicates.

Gram-Positive Strains	A3	Parent Peptide
*S. aureus* (29213)	25	No activity
*S. aureus* (BAA-41)	30	No activity

**Table 7:** Percentage reduction of viable bacterial cells of *S. aureus* biofilms including the minimum biofilm bactericidal concentration (MBCb) for A3 against tested bacterial strains.

Peptide Conc. (µM)	80	60	40	35	30	25	20	15
*S. aureus* (29213)	0.028%	0.083%	0.13%	0.24%	0.46%	5.7%	11.6%	25.3%
*S. aureus* (BAA-41)	0.013%	0.098%	1.2%	2.6%	4.7%	13.2%	27.4%	35.2%

**Table 8:** IC_50_ values for A3 on HEK and Vero mammalian cell lines. Results represent triplicates.

A3	**Mammalian IC_50_ (µM)**	
	**HEK**	**Vero**
	33.2	26.1

**Table 9:** Hemolytic effect A3 on human erythrocytes after 60 min of incubation. Results represent triplicates.

Peptide Concentration (µM)	Hemolysis (%)
1	0
5	0
10	5.1
20	16.8
40	36.1
60	47.9
80	49.4

## Supplementary Materials

Supplementary Materials are available online. Figure S1: Analytical RP-HPLC chromatogram of **A3**, Figure S2: Positive electrospray ionization (ESI) mass spectrometric (MS) analysis of the **A3**. The peptide showing major peaks in the +2 and+3 charge state of 991.64 Da and 661.37 Da respectively.
