# Supplementary material for: A3, a Scorpion Venom Derived Peptide Analogue with Potent Antimicrobial and Potential Antibiofilm Activity against Clinical Isolates of Multi-Drug Resistant Gram Positive Bacteria
Source: Molecules. 2018 Jul 2;23(7):1603. doi: 10.3390/molecules23071603 (PMC6100099; doi:10.3390/molecules23071603)
Supplement: Supplementary file 1 [file molecules-23-01603-s001.pdf]

A3, a Scorpion Venom Derived Peptide Analogue with Potent Antimicrobial and Potential Antibiofilm Activity against Clinical Isolates of Multi-Drug Resistant Gram Positive Bacteria

Supplementary Material:

HPLC REPORT

Product Name:FK-18
Instrument No: 0200194
Lot No :P150414-SL343091
Column :4.6*250mm, phenomenex C18-5
Solvent A :0.1%Trifluoroacetic in 100% Acetonitrile
Solvent B :0.1%Trifluoroacetic in 100% Water

Gradient :	A	B
0.01min	48%	52%
25in	73%	27%
25.1min	100%	0%
30min	Stop	

Flow rate :1.0ml/min
Wavelength :220nm
Volume: 10µl

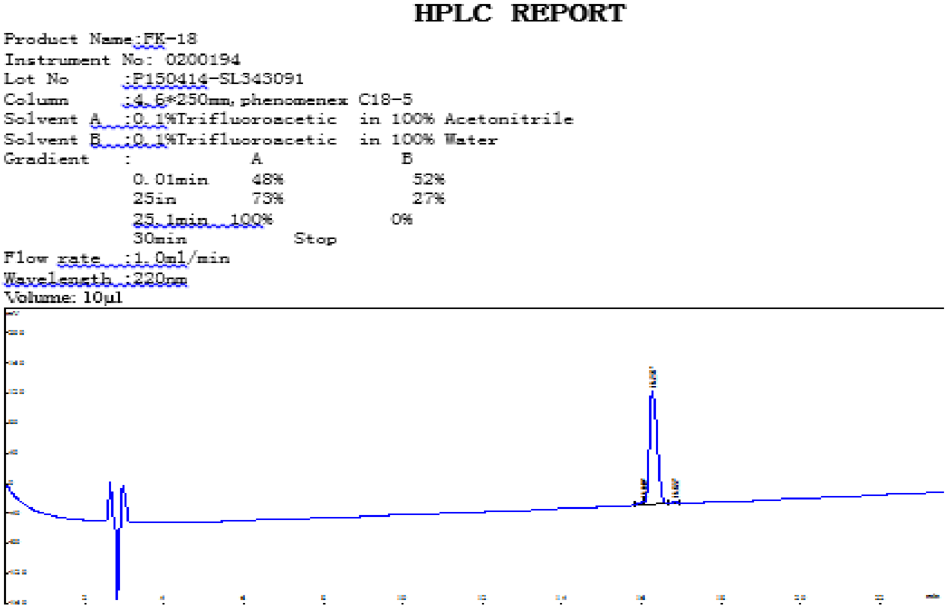

Rank	Time	Name	Conc.	Area	Height
1	16.049		1.618	29090	3700
2	16.258		97.55	1754252	150172
3	16.822		0.829	14907	1504
Total			100	1798249	155376

Figure S1. Analytical RP-HPLC chromatogram of A3

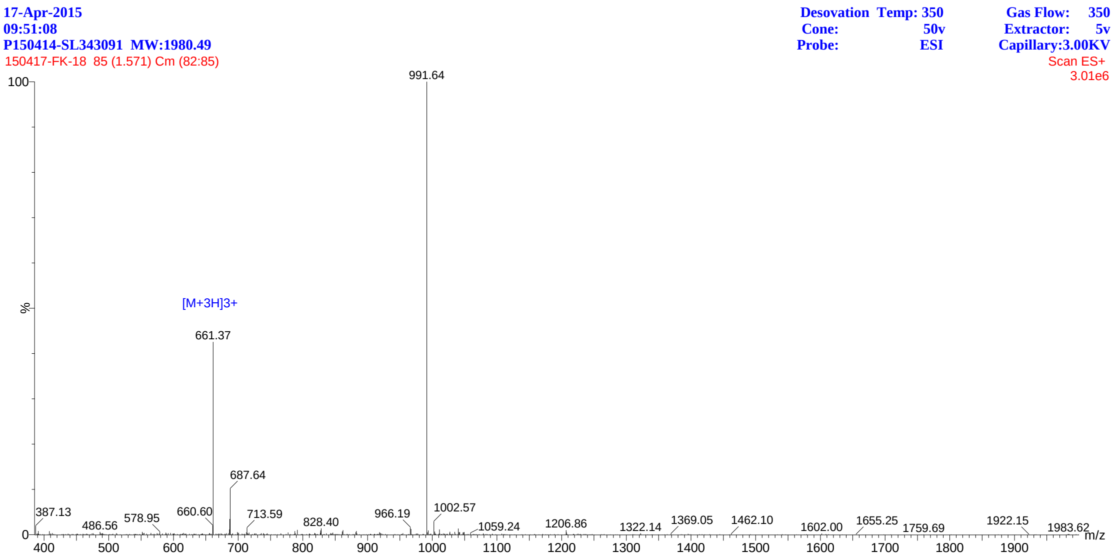

Figure S2. Positive electrospray ionization (ESI) mass spectrometric (MS) analysis of the A3. The peptide showing major peaks in the +2 and+3 charge state of 991.64 Da and 661.37 Da respectively
